# Development and preliminary physicochemical characterization of a Ramucirumab biosimilar candidate in CHO cells using shake-flask fed-batch culture

**DOI:** 10.1007/s00449-026-03358-y

**Published:** 2026-05-27

**Authors:** Elcin Cagatay, Yonca Gungor, Sadettin S. Ozturk, Hulya Ayar Kayali

**Affiliations:** 1 Izmir International Biomedicine and Genome Institute, Dokuz Eylul University, Izmir, Turkey; 2 Izmir Biomedicine and Genome Center, Izmir, Turkey; 3 OzBio LLC, Dedham, MA USA; 4 Department of Chemistry, Faculty of Science, Dokuz Eylul University, Izmir, Turkey

**Keywords:** CHO cells, Monoclonal antibody (mAb) production, Physicochemical characterization, VEGFR2, Ramucirumab, Biosimilar

## Abstract

The development of biosimilar monoclonal antibodies requires strict control of critical quality attributes and a detailed understanding of cell culture performance. This study describes the development and preliminary physicochemical characterization of a candidate biosimilar monoclonal antibody targeting VEGFR-2 produced in CHO cells. Seventy-five clones generated through gene transfection were screened based on productivity and cell viability. Culture parameters including pH, glucose concentration, cell growth, and cell specific productivity (Qp) were monitored to evaluate their influence on production performance. Eight high producing clones were selected for shake-flask scale fed-batch production. The lead clone achieved a final titer of 2.79 g/L. The biosimilar candidates were characterized using intact mass spectrometry, size exclusion chromatography, cation exchange chromatography, hydrophobic interaction chromatography, glycan analysis, and peptide mapping. Binding affinities were evaluated using SPR, demonstrating comparable interaction with VEGFR-2. The analytical results demonstrated structural similarity to the reference product with no critical differences in purity, charge variants, glycosylation patterns, or binding affinity. These findings demonstrate the importance of systematic clone screening and upstream process monitoring for achieving consistent product quality in biosimilar antibody production and provide practical insights for mammalian cell-based bioprocess development.

**Supplementary Information:**

The online version contains supplementary material available at 10.1007/s00449-026-03358-y.

## Introduction

Monoclonal antibodies (mAbs) are laboratory produced molecules designed to bind tumor specific or tumor associated antigens and restore, enhance, or modulate immune mediated anti-cancer responses [4]. Owing to their high target specificity and favorable safety profiles, mAbs represent one of the most successful classes of biopharmaceutical therapeutics [27]. As of 2024, approximately 200 monoclonal antibodies have been approved for marketing in different parts of the world [21]. However, the development of novel mAbs remains a time consuming and resource intensive process due to high production costs, complex manufacturing requirements, and extensive clinical validation. Consequently, the development of biosimilars that are highly similar versions of already approved products has emerged as a strategic approach to improve accessibility and reduce healthcare costs (Sekhon & Salujja, 2011).

Biosimilar development follows a stepwise “totality of evidence” approach aimed at demonstrating similarity in quality, safety, and efficacy to the reference product. At each stage, extensive analytical and functional comparisons are performed to minimize residual uncertainty and guide subsequent head-to-head evaluations. In contrast to novel biologics, where clinical trials constitute the primary basis for approval, biosimilar development relies predominantly on comprehensive analytical characterization supported by limited non-clinical and clinical studies [6]. This approach is well established within regulatory frameworks and is reflected in guidance documents provided by key health authorities such as the U.S. Food and Drug Administration (FDA), the European Medicines Agency (EMA), and the World Health Organization (WHO) [23].

Vascular endothelial growth factor receptor-2 (VEGFR-2) is a central mediator of pathological angiogenesis, where its activation initiates various cellular pathways contributing to tumor progression and metastatic spread [29]. VEGFR-2 targeting mAbs, like Ramucirumab, continue to be very relevant biosimilar targets within this regulatory framework. As of early 2026, no biosimilar version of Ramucirumab had received approval in significant markets like the US or the EU, despite the increasing number of approved biosimilars worldwide. Given its clinical importance in oncology and the continued absence of biosimilar competition, the development of Ramucirumab biosimilars represents a valuable opportunity to improve treatment accessibility while addressing unmet needs in cost-effective biologic therapies.

The intrinsic structural complexity and post-translational heterogeneity of mAbs render their production highly process-sensitive, requiring precise control and optimization of both upstream and downstream operations to ensure batch-to-batch consistency. Although the literature extensively addresses analytical similarity assessments, systematic investigations focusing on the optimization of the complete manufacturing pipeline remain relatively limited. In this work, we present the development and initial assessment of an upstream and downstream process for Ramucirumab, a putative VEGFR-2-targeting biosimilar candidate, encompassing gene design, upstream process optimization, downstream purification, and initial structural characterization.

## Materials and methods

### Gene design

The heavy and light chain amino acid sequences of Ramucirumab used in this study are provided in the Supplementary Materials. Coding sequences were generated using the EMBOSS Backtranseq tool with the codon usage table of *Cricetulus griseus* to optimize expression in Chinese Hamster Ovary (CHO-K1) cells. A Kozak consensus sequence was incorporated upstream of the start codon to enhance translational efficiency. Each gene was preceded by a signal peptide sequence to facilitate secretion of the recombinant antibody. Appropriate restriction enzyme recognition sites were introduced for directional cloning into a glutamine synthetase (GS) based dual-expression vector backbone. The heavy and light chain constructs were co-expressed within the same plasmid to enable coordinated antibody assembly. Plasmid DNA was linearized using PvuI restriction enzyme and purified by ethanol precipitation. Briefly, 3 M sodium acetate (pH 5.2) and ice-cold ethanol were added, followed by incubation at − 80 °C for 2 h. Samples were centrifuged and the pellet was resuspended in TE (10 mM Tris-HCl, 1 mM EDTA, pH 8.0 buffer.

### Stable cell line development

The CHO K1 cells were used as host cells for the generation of stable cell lines. CD CHO AGT serum-free media (Gibco, Cat: 12490-025) supplemented with 6 mM L-glutamine (Gibco, Cat: 25030-024), Pluronic F68 (Gibco, Cat: 24040-032) and HT supplement (Gibco, Cat: 11067-030) were used to grow the suspension cells. The cells were grown in 125 mL shake flasks (Corning, USA) on a Celltron shaker (Infors HT, Switzerland) at 140 rpm at 37 °C with 8% CO_2_ and 88% humidity. The cells were passaged every 3‒4 days at a density of 2‒3 × 10^5^ cells/mL.

Transfection into the CHO K1 host cells was carried out via the Lonza 4D Nucleofector device and Amaxa SG Cell Line 4D-Nucleofector X kit (Lonza Bioscience, Cat: V4XC-2024). Transfected cells were selected via 50 µM methionine sulfoxamine (MSX, Sigma Aldrich, Cat: M5379). Single-cell cloning was performed using different basal media supplemented with HT supplement, GS supplement, conditioned medium, and phenol red either with or without MSX. Cells were seeded at 0.5 cells/well in 96-well plates and incubated at 37 °C with 5% CO₂ and 88% humidity for 2–3 weeks. Proliferating clones were picked and expanded for subsequent fed-batch culture.

### Production and purification

The fed-batch production involved a 14-day culture period, employing two distinct media formulations, Media A (GM_A) and Media B (GM_B), combined with four separate feed additions. In accordance with the varying viable cell densities (VCDs) and culture volumes each day, a daily supplement of 2–3% feed was administered to the cultures.

Following the manufacturer’s instructions, a 1 mL HiTrap mAbSelect PrismA (GE Healthcare, CA) column that had previously been equilibrated with PBS was used to purify the product. 5 mL of the conditioned media was loaded to the column, and eluted with 4 mL 0.1 M citrate buffer, pH 3, in 300 µl 0.5 M NaOH, pH 9. The purified mAbs were stored at -20 °C until further analysis.

For the quantification of product concentration in cell culture supernatant, enzyme linked immunosorbent assay (ELISA) was performed. Detailed protocol is provided in the Supplementary Materials. The total protein concentration was assessed by measuring the absorbance at 280 nm and with BCA. For spectrophotometric measurement, NanoDrop™ 2000/2000c Spectrophotometer (Thermo Fisher Scientific, Waltham, MA) was used. Total protein was assessed with a Pierce BCA protein assay kit (Thermo Fisher Scientific, Cat: 23225) according to the manufacturer’s instructions. Qp values were calculated according to the study by Paul et al. [25].

### Quality control and characterization

For the characterization and comparison of the produced mAbs with the reference molecule, gold standard methods such as sodium dodecyl sulfate‒polyacrylamide gel electrophoresis (SDS-PAGE), size exclusion chromatography (SEC), intact mass analysis, peptide mapping, glycosylation assessment, hydrophobic interaction chromatography (HIC), cation exchange chromatography (CEX) and surface plasmon resonance (SPR) were performed. Detailed protocols used in this study are provided in the Supplementary Materials.

## Results

**Fig. 1:**
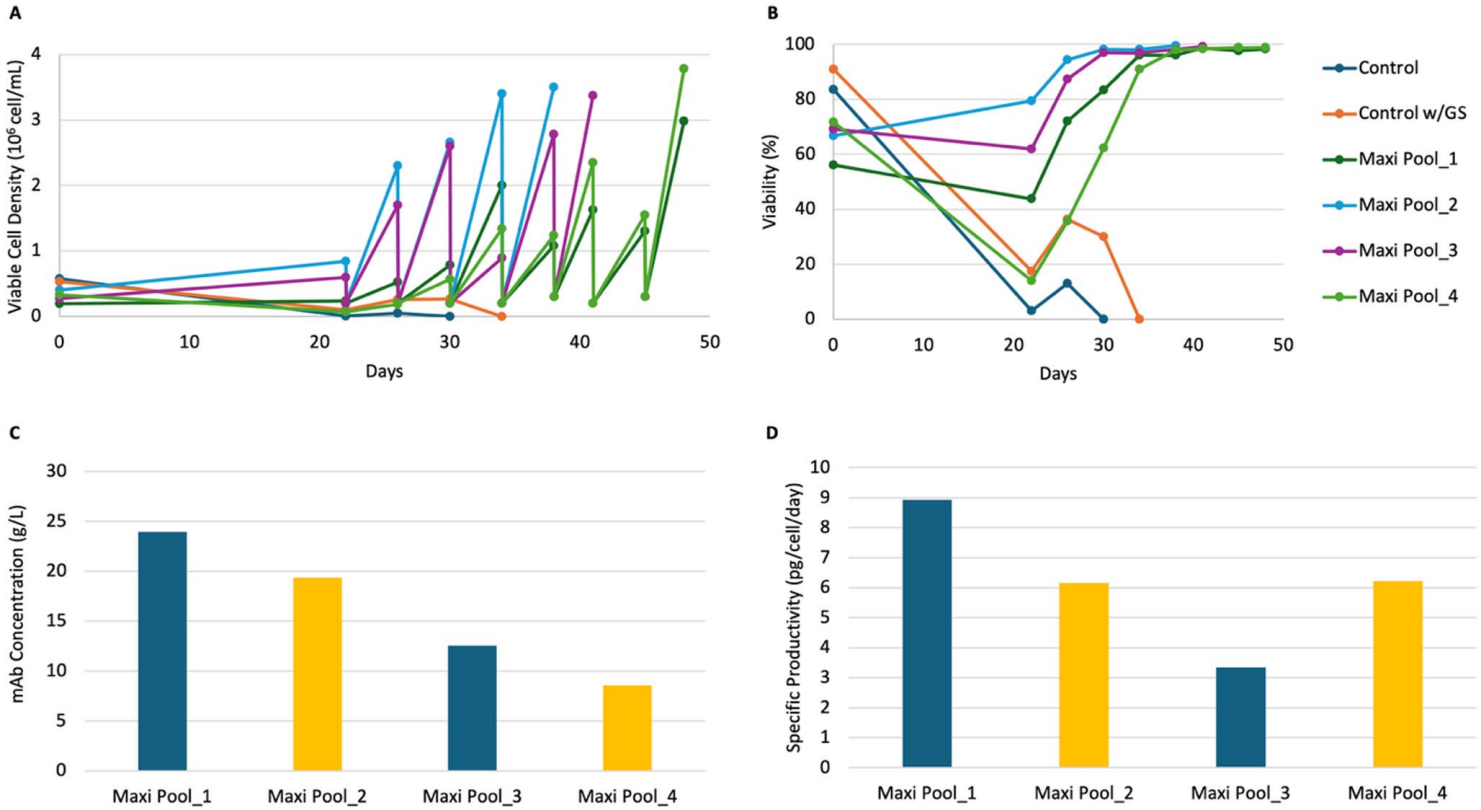
Cultivation of recombinant maxi pools (*n* = 1 for each condition). Maxi pools_2 and 4 were supplemented with GS, and Maxi pools_1 and 3 were cultured without GS supplementation. **A** Measured VCDs and **B** % viability of the maxi pools, **C** concentration of produced mAb measured by ELISA, **D** specific productivity of each maxi pool

The VCD and viability data for the host cell line are shown in Fig. S1. The host CHO K1 cells exhibited a repeated growth pattern driven by routine seeding steps, while maintaining high viability (> 90%) throughout the culture period. The growth pattern data for the recombinant maxi pools are presented in Fig. 1A and B. The results demonstrate that maxi pool cultures exhibit significantly improved growth and viability profiles compared to control groups. In control groups, VCD rapidly declined after day 20, reaching near-zero levels by approximately day 30–35. In contrast, Maxi Pool groups demonstrated a marked increase in VCD after day 25, with peak values observed between days 30 and 48. Consistent with VCD results, cell viability in control groups decreased sharply, dropping below 20% by day 25 and reaching 0% by day 30. In contrast, Maxi Pool groups initially showed a moderate decline in viability but subsequently recovered, maintaining high viability levels (approximately 90–100%) until the end of the culture period. Figure 1C shows the mAb concentrations obtained from the maxi pools. The highest Qp value was determined to be 8.92 pg/cell/day in Maxi pool_1. In Maxi pool_2, Maxi pool_3 and Maxi pool_4, the Qp values were 6.16, 3.34, and 6.22, respectively (Fig. 1D).

**Fig. 2:**
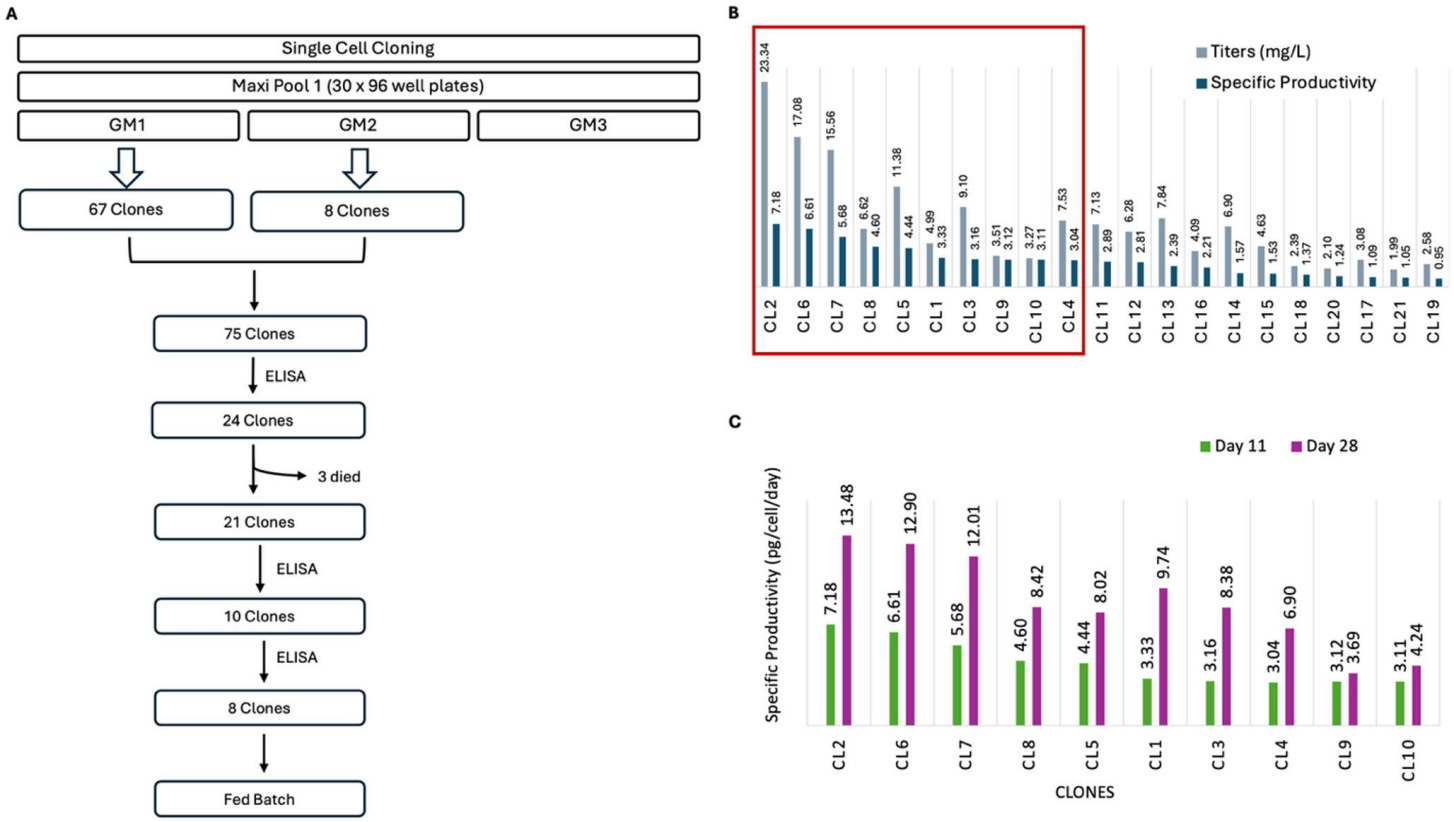
Summary of the single-cell cloning process. **A** Flow chart of the single-cell clone selection process **B** Titers measured on day 11 after picking the clones from the single-cell cloning experiment. Specific productivity was calculated, and the 10 clones with the highest values were selected for further growth **C** On days 11 and 28, the titer of each cell clone was measured via ELISA

The highest producing maxi pool, Maxi pool_1, was considered suitable for single-cell cloning. The flow chart of the single-cell clone selection process is summarized in Fig. 2A. The Qp values of the highest 24 clones were in between 0.95 and 7.18 pg/cell/day.

During cultivation, three clones lost their viability, and the studies were carried out with 21 clones, of which the titer and Qp data are shown in Fig. 2B. We further selected the ten clones with the highest yield. During the routine cultivation period all clones showed comparable growth trends during the early phase, whereas notable differences emerged at later time points with some clones reaching significantly higher maximum cell densities (Fig. S2A). The Qp data for days 11 and 28 are presented in Fig. 2C. On day 11, the Qp ranged from 3.11 to 7.18 pg/cell/day, whereas on day 28, it ranged from 4.24 to 13.48 pg/cell/day. There was a decrease in the viability of each clone between days 11 and 28 (Fig. S2B). To recover the cells from the growth-inhibited state and prepare them for fed-batch culture, we replaced CD CHO media with a richer media, GM_A media, on day 32. Following media replacement, all the cell clones reached a viability greater than 92%, and the maximum doubling time was 0.8 days.

**Fig. 3:**
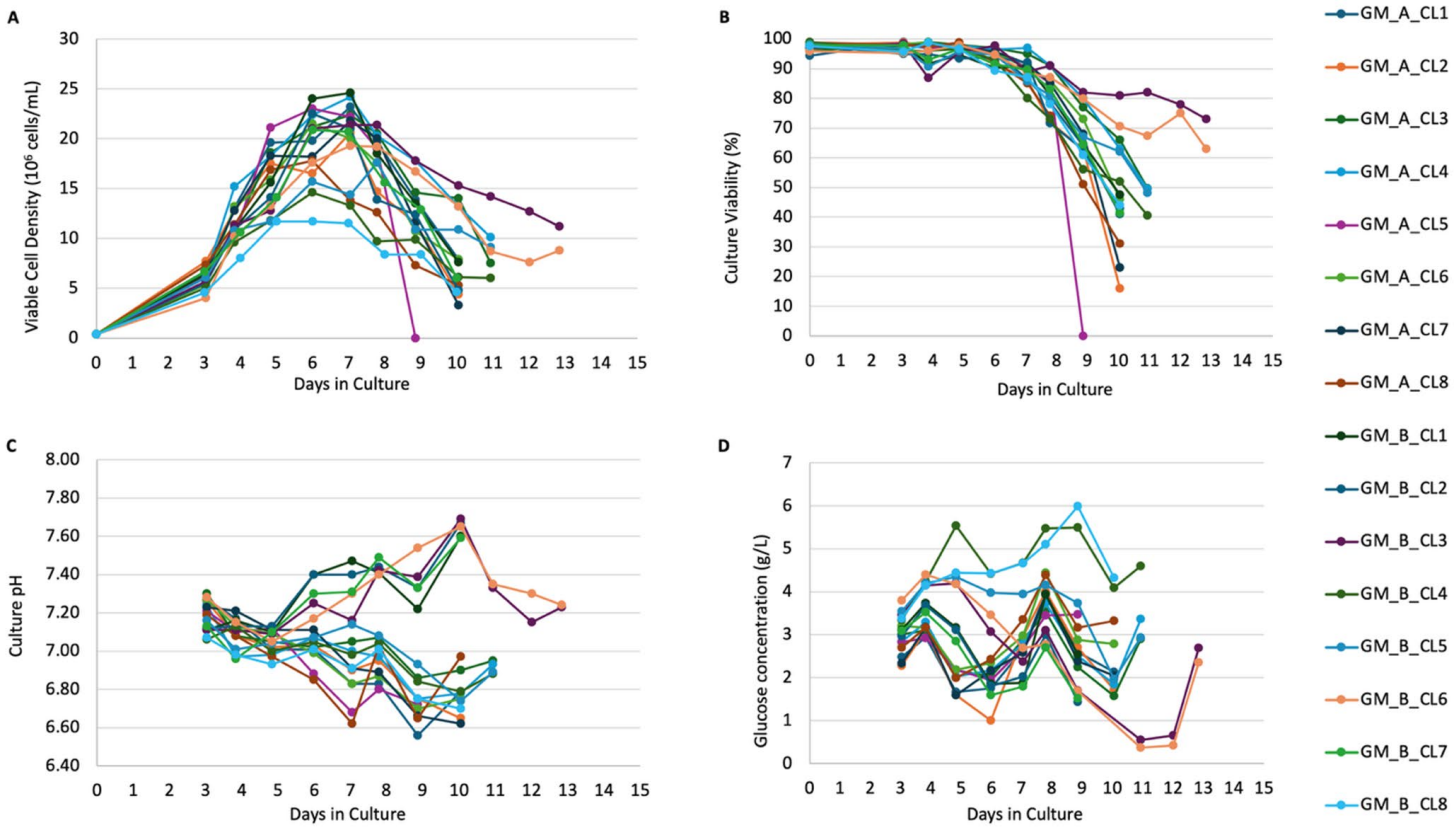
Fed-batch culture process with the 8 selected pools with two different types of cell culture media (*n* = 1 for each clone). **A** VCD, **B** culture viability percentage, **C** culture pH, and **D** glucose concentration (g/L) were measured every day starting from day 3

Throughout the fed-batch culture the maximum VCDs ranged between 17.8 and 24.2 × 10^6^ cells/mL for GM_A and between 11.7 and 24.6 × 10^6^ cells/mL for GM_B (Fig. 3A). As shown in Fig. 3B, the viability of the clones from day 0 to day 6 remained above 95%, whereas the overall viability of the cultures started to decrease from the fifth day onward. Among them, GM_A_CL5 was the first culture to lose all the viable cells on the ninth day. For the rest of the cultures, the VCDs and viabilities started to decrease in general from the seventh day onward during the production process. The pH of the GM_A cultures ranged from 6.56 to 7.30, whereas it ranged from 6.74 to 7.60 for the GM_B cultures.

The glucose levels in the GM_A cultures decreased below the critical level of 2 g/L for all the clones on day 5, whereas those in the GM_B cultures remained above this level until day 6. Clones GM_B_CL3 and GM_B_CL6, which completed the 14-day fed-batch process with high viability and titers, decreased below the critical value on the 9th day.

The titers before purification reached a maximum of 2.4 g/L (Table S1). The cells cultured in GM_A had titers ranging from 0.94 to 1.42 g/L, whereas the cells cultured in GM_B had titers ranging from 0.92 to 2.4 g/L. The supernatants were then purified manually using HiTrap mAbSelect PrismA column (GE Healthcare, CA). The titers of the purified mAbs were measured via a BCA and UV280 assays (Table S2). GM_B_CL clones showed variability in product levels across both methods. The highest titers were observed in GM_B_CL_3 (2.79 g/L by BCA; 2.64 g/L by UV280) and GM_B_CL_6 (2.46 g/L; 2.17 g/L), indicating higher productivity compared to the remaining clones. Mid-range productivity levels were obtained for GM_B_CL_2 and GM_B_CL_7. The purification yield was calculated by dividing the eluted protein amount to the loaded protein amount and it ranged between 29.9 and 91.37%. Clones GM_B_CL_2, GM_B_CL_3, and GM_B_CL_6 exhibited the highest Protein A recovery yields (68–91%) among all clones.

SDS-PAGE analysis showed that most of the contaminant proteins were successfully removed by Protein A purification (Fig. S3). In Fig. S3B, sample and reference bands align, whereas in Fig. S3E, the reference band runs slightly lower than the mAbs. It should be noted that the same reference product was used across all panels. Therefore, the difference in Fig S3E may be influenced by sample handling, loading conditions, or formulation components present in the reference product. For the reduced purified samples (Fig. S3C and Fig. S3F), the sample bands were perfectly aligned with those of the reference.

**Fig. 4:**
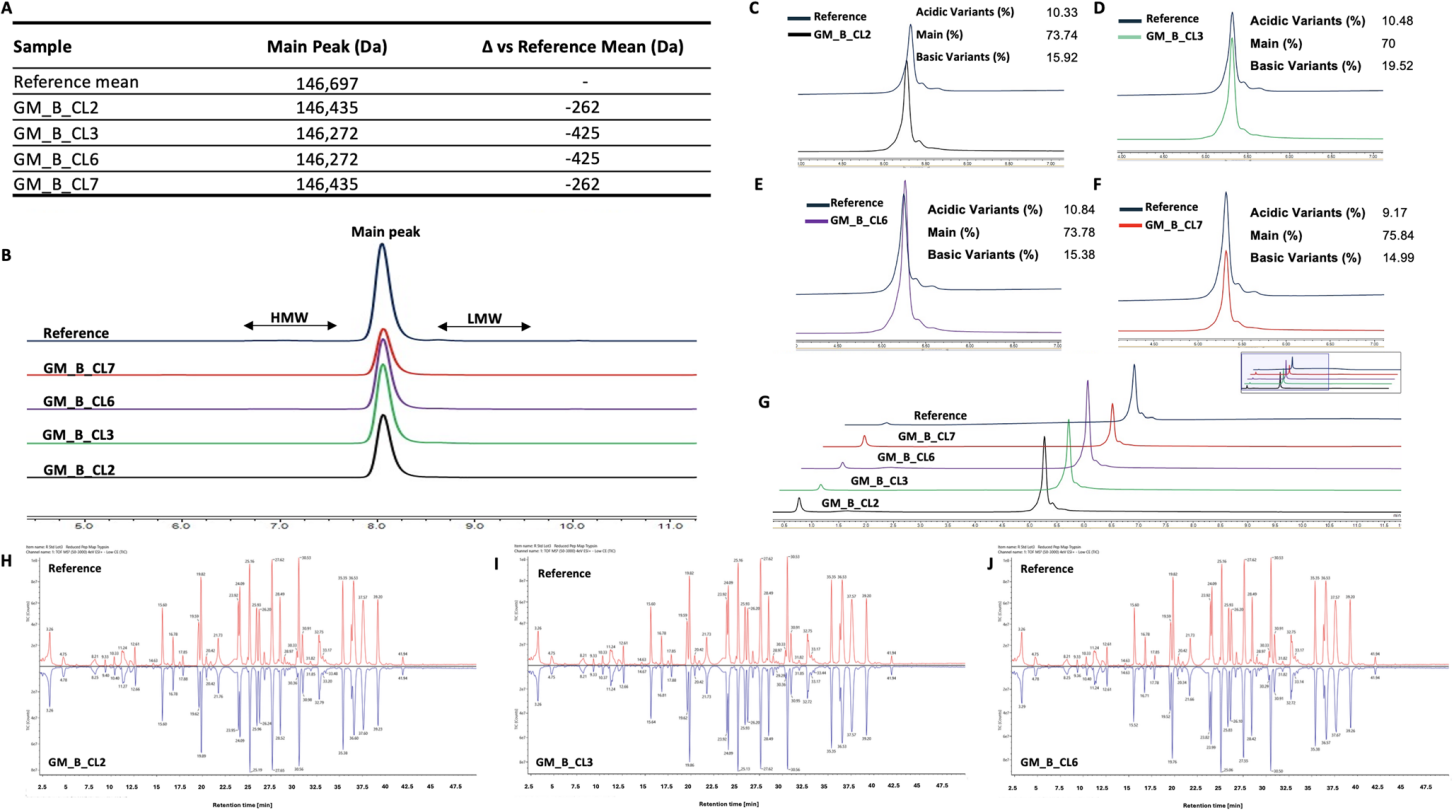
Physicochemical characterization of reference and GM_B clones. **A** Intact mass analysis of reference and GM_B clones, showing main peak masses and mass differences (Δ) relative to the reference mean **B** SEC profiles of reference, GM_B_CL2, GM_B_CL3, GM_B_CL6, and GM_B_CL7 showing monomer, HMW and LMW species. **C**–**F** CEX chromatograms of GM_B_CL2, GM_B_CL3, GM_B_CL6, and GM_B_CL7, respectively, compared to the reference, indicating charge variant distribution (acidic, main and basic peaks). **G** Overlay of CEX profiles for all samples. (H–J) Peptide mapping profiles of reference, GM_B_CL3, GM_B_CL6, and GM_B_CL7, confirming comparable sequence coverage

The mass spectrometry spectra demonstrated a high degree of similarity between the reference lots and the GM_B_CL samples (Fig. 4A, Figure S4). The main peak of the reference material was determined as 146,697 Da. In comparison, all GM_B clones exhibited lower molecular masses, with shifts of − 262 Da (GM_B_CL_2 and GM_B_CL_7) and − 425 Da (GM_B_CL_3 and GM_B_CL_6).

SEC analysis showed a strong similarity to the reference (Fig. 4B). The reference was detected at 8.046 min, while GM_B_CL3 and GM_B_CL6 were detected at 8.054 min, and GM_B_CL2 and GM_B_CL7 at 8.063 min. No high-molecular-weight (HMW) peaks were observed. However, all clones displayed a low-molecular-weight (LMW) peak at 11.563 min, which was not present in the reference.

For CEX analysis, different lots of the reference are analyzed to provide a reference range for the clonal mAbs (only one of them is shown in the chromatograms). There were three distinct chromatographic peaks observed in the reference mAb chromatograms: the area of the acidic peak was between 7.35% and 10.8%, the major peak area was 62.81% and 72.98%, and the basic peak area was 16.12% and 29.85%. The percentages of the acidic variants of GM_B_CL2 (Fig. 4C), GM_B_CL3 (Fig. 4D) and GM_B_CL7 (Fig. 4F) were in line with the reference values, whereas CM_B_CL6 (Fig. 4E) had a slightly greater percentage. The basic variant of GM_B_CL3 (Fig. 4D) was inside the reference range, whereas other clones had slightly lower percentages. In addition to the main analytical data, an overlay of the CEX profiles for all samples is presented in Fig. 4G, allowing direct comparison of charge variant distributions.

Fragmented peptide masses of the reference and most similar three clones selected in previous analyses, GM_B_CL2, GM_B_CL3, and GM_B_CL6, showed highly similar fragmentation patterns (Fig. 4H and J). Minor differences in oxidation and deamidation patterns were detected between the samples and the reference.

**Fig. 5:**
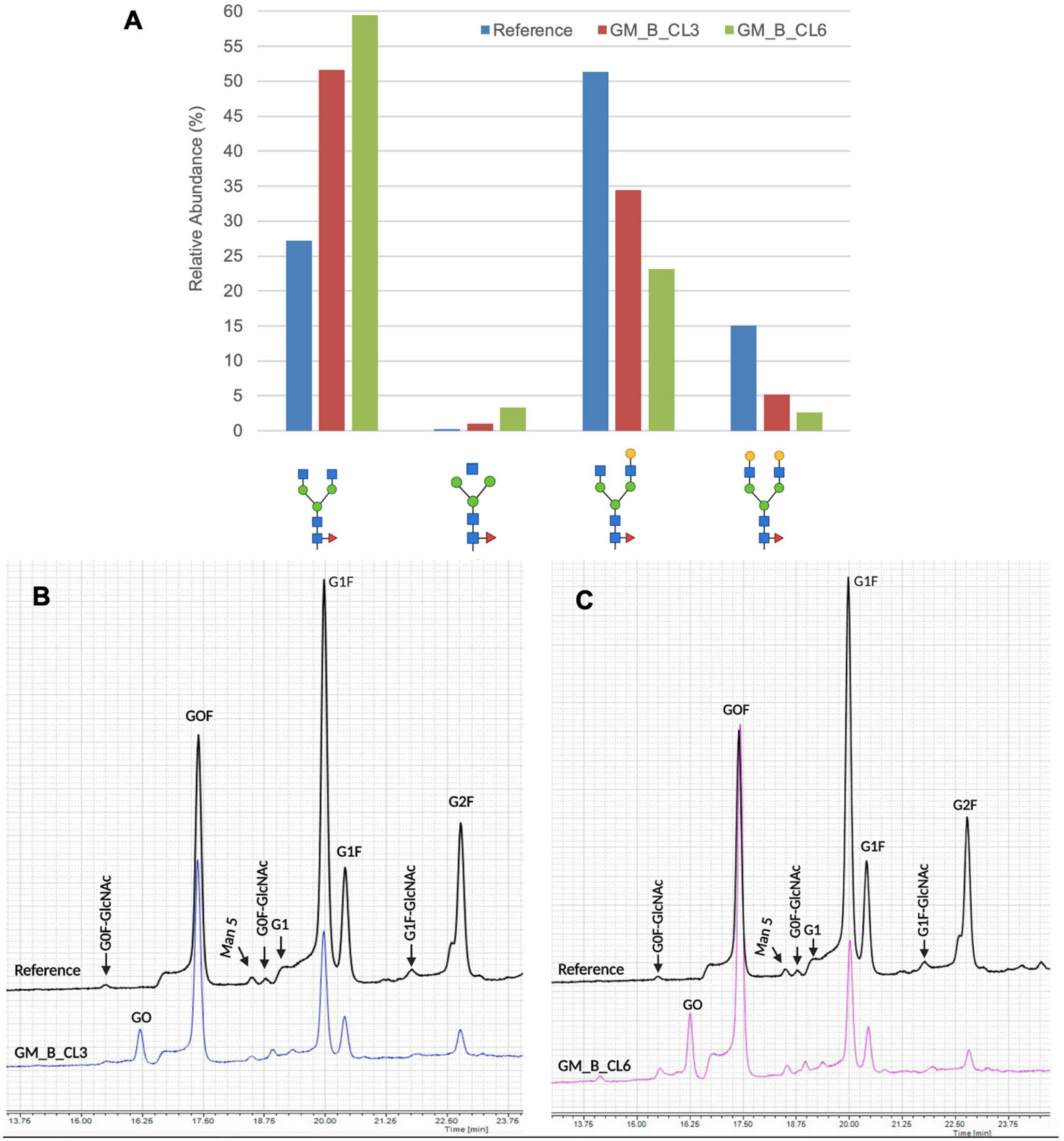

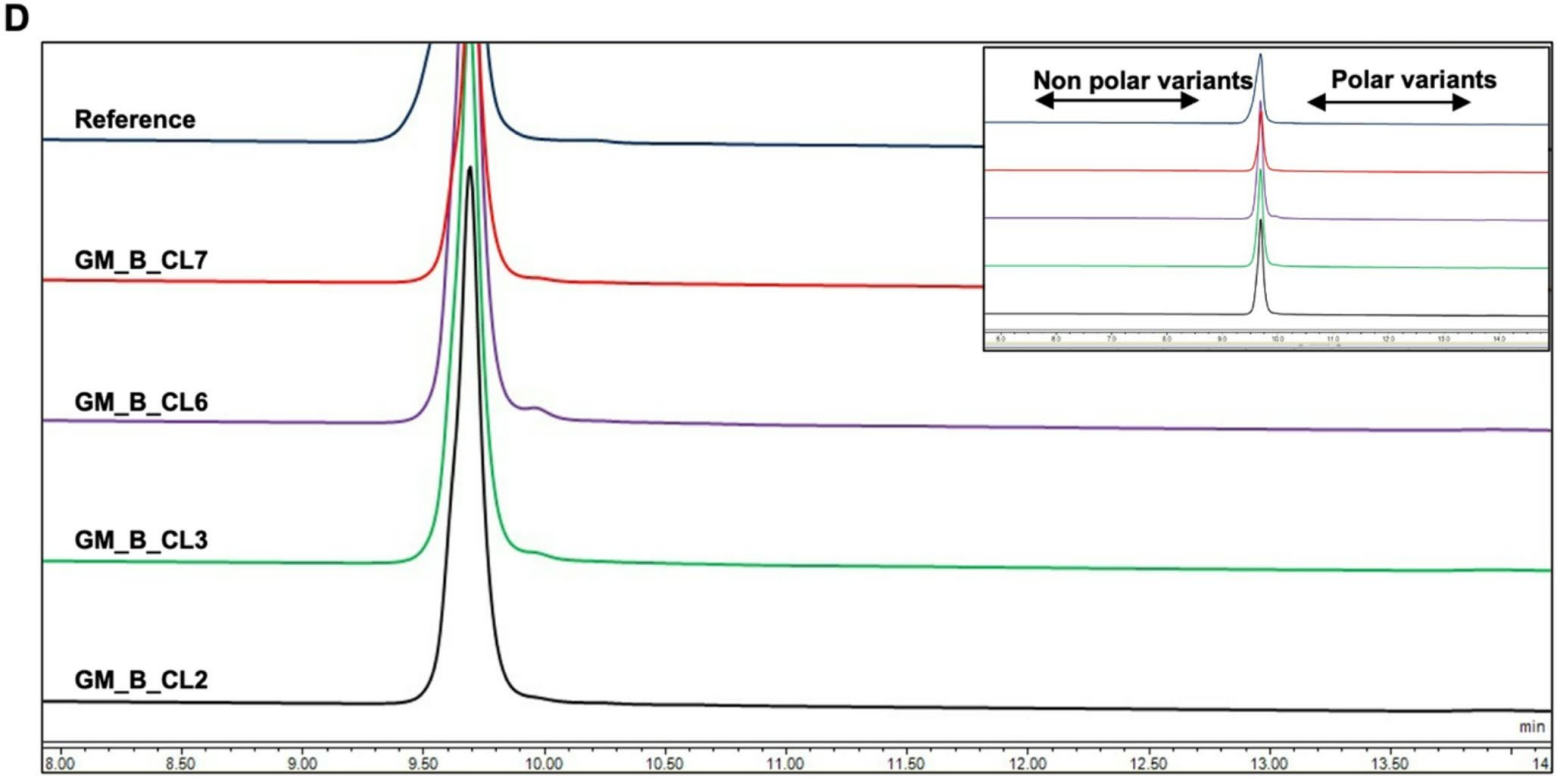
Glycosylation patterns and HIC chromatograms of the reference and produced mAbs. **A** Fucosylated glycoform percentages, comparison of LC chromatograms with reference and **B** GM_B_CL3 and **C** GM_B_CL6 samples **D** HIC chromatograms for the samples obtained from the GM_B fed-batch cultures

Eight glycoforms in total were determined in the samples. We observed differences in the glycoform populations identified for each mAb; afucosylated glycans constituted 5.32% of the reference, 7.46% of GM_B_CL3 and 11.08% of GM_B_CL6. Among the major fucosylated glycoforms, G0F and G1F were the most abundant in both the reference and produced mAbs (Fig. 5A). In line with the results of mass spectrometry, also HPLC analysis demonstrated that G0F and G1F were the most abundant glycan structures in the produced mAbs (Fig. 5B and C). The percentage of the high-mannose (Man5) glycoform population was determined to be 0.47% for the reference, 0.9% for GM_B_CL3, and 1% for GM_B_CL6. The Man5 structure is present at very low levels in both the reference and produced mAbs.

The HIC profiles demonstrated a high degree of similarity, except that the mAbs purified from the GM_B_CL6 culture presented a small peak following the main peak (Fig. 5D).

Table S3 presents the binding affinities (Kd value) of the produced mAbs and the reference. The binding affinities of different lots of the reference ranged between 10.55 and 32.56 pM. Among the produced mAbs, GM_B_CL2 and GM_B_CL6 showed Kd values of 29.67 and 34.95 pM, respectively. GM_B_CL3 exhibited a lower Kd of 7.17 pM, indicating higher affinity, whereas GM_B_CL7 demonstrated the lowest binding affinity for VEGFR-2, with a Kd value of 82.37 pM.

## Discussion

This study presents an early-stage assessment of a production and analytical workflow for a proposed Ramucirumab biosimilar candidate. Overall, the results suggest initial analytical similarity to the reference product, while highlighting variability among clones in terms of production and purification efficiency.

One commonly examined physiological parameter in recombinant cell culture processes is specific productivity (Qp), which is a crucial parameter for comprehending the connections between cell physiology and product quality [28, 33]. The highest Qp value was determined in Maxi pool_1, which was transfected in the absence of GS supplementation. The addition of GS supplement contributed to cell growth even in the first days after transfection but led to the formation of pools with lower production (Fig. 1C). GS supplementation provides amino acids such as alanine, asparagine, aspartic acid, glutamic acid, proline, and serine as well as the nucleosides adenosine, guanosine, cytidine, uridine, and thymidine. Although nucleoside supplementation was shown to improve growth without reducing Qp in previous studies [30], amino acid supplementation may favor MSX selection, consequently decreasing specific productivity while improving growth overall. In addition, high serine levels in the media increase glucose consumption and consequently lactate production, which may be a factor limiting productivity [16].

Taking the day of clone collection as day 0, an increase in the Qp values of all clones was observed by day 28. This increase in Qp can be attributed to several factors. First, as Al-Rubeai et al., [3] suggested, cells in the G1/early S phase tend to allocate their energy resources more toward mAb synthesis rather than proliferation [3]. Notably, slower-growing cells presented significantly higher productivity. This observation is consistent with our study, where the doubling times of 7 out of 10 clones were almost doubled from the 11th day to the 28th day, potentially explaining the increase in productivity (Fig. S2C). Second, over the 18-day period, the average viability of the cells decreased by 22% (Fig. S2B). This loss of viability may have contributed to the release of mAbs upon cell death, which could have been actively retained when they were viable, resulting in an apparent increase in Qp. The observed decrease in viability between days 11 and 28 can be attributed to the prolonged growth-inhibiting state of the cells [3]. Third, Pluronic F-68 was used in the cultures, a copolymer surfactant, to protect the recombinant cells from shear stress [13]. Previous studies have shown that the use of Pluronic F-68 in cultures increases the productivity of hybridoma cells as well as CHO cells [2, 5]. The combination of these factors likely contributed to the observed increase in productivity during routine passages (Fig. S2D).

During the fed-batch process, the glucose levels in the cultures started to decrease to the critical level of ~ 2 g/L on the 5th day. These cultures were terminated on the 8th-11th days because of low viability. In GM_B_CL3 and GM_B_CL6, which completed the 14-day fed-batch process with high viability and titers, a decrease below the first critical value of 2 g/L occurred on the 9th day. The subsequent decrease in viability due to glucose consumption can be attributed to the byproducts produced throughout the culture process, which consequently impact the pH of the cultures [19]. However, lactate and ammonia levels were not monitored in this study due to analytical scope limitations, which limits the ability to directly correlate metabolite accumulation with the observed pH changes, viability decline, and productivity trends.

In the biopharmaceutical industry, immunogenicity is a critical factor for the approval of biosimilar mAbs, with the presence of aggregates being a key contributor to unwanted immune responses [10]. Aggregates can form during production, storage, or delivery; therefore, monitoring the aggregation profile throughout these stages is essential. In this study, SDS-PAGE and SEC analyses revealed purity and size consistency across all clones when compared to the reference.

In the non-reducing SDS-PAGE image (Fig. S3E), slight differences in molecular weight between the reference and the produced mAbs, as well as the presence of additional lower bands, may indicate incomplete denaturation [18]. A faint band observed around 100 kDa could be a fragmentation artifact of the two heavy chains, potentially caused by components in the loading dye [35]. Conversely, in the reducing SDS-PAGE gel (Fig. S3C), although full reduction appears to be achieved, the presence of multiple bands for each chain may reflect heterogeneity in denaturation. Furthermore, Fig. S3F reveals two extra bands around 125 kDa and 75 kDa, which are likely artifacts of incomplete reduction, corresponding to 2 heavy + 1 light chain (2HL) and 1 heavy + 1 light chain (HL) fragments [35].

Intact mass analysis revealed that all GM_B_CL clones displayed a primary species that closely aligned with the reference lots (~ 146.2–146.4 kDa), suggesting that their structural integrity was largely maintained. The observed minor mass shifts (− 200 to − 425 Da) were also within the variability range of the reference material, suggesting that these differences likely reflect typical product heterogeneity rather than significant structural modifications or degradation. In SEC analysis, all clones displayed a dominant monomeric peak, accompanied by a low molecular weight species that was not observed in the reference material. Literature reports that LMW species observed in SEC analyses may arise from multiple mechanisms, including hinge region cleavage [8], disulfide bond heterogeneity [32], and conformational or proteolytic truncation producing complementary fragments [31] although the exact contribution of each pathway is often product- and process-dependent and cannot be definitively assigned without orthogonal analytical evidence. In addition, both storage conditions and upstream/downstream process parameters are recognized as key drivers of LMW formation, as temperature, pH, freeze–thaw stress (Abdelghaffar et al.,2022), and manufacturing-related redox [32] or cell culture conditions [7] can significantly influence LMW levels.

Charge heterogeneity is a critical quality attribute for monoclonal antibodies, as it can influence their pharmacokinetics, stability, and immunogenicity [17]. All clones exhibited acidic variants within the reference range, suggesting no significant increase in degradation-related variants such as deamidated species. The basic peaks also aligned closely in both position and intensity, indicating a high degree of structural consistency. Among the clones, GM_B_CL2 and GM_B_CL6 demonstrated the highest similarity to the reference in terms of charge variant profiles, further supporting their biosimilarity. These results reinforce the comparability of the produced mAbs and suggest that the upstream processing and purification conditions were well-controlled, minimizing charge-altering modifications.

The primary structure of the produced mAbs was analyzed via mass spectrometry. Although comparison of fragmented peptide masses alone is insufficient to definitively identify specific modifications [20], a high degree of similarity was observed between the reference and the mAb samples, despite minor differences in the chromatograms. Nevertheless, considering these results alongside other analyses, the observed disparities were deemed insignificant and unlikely to affect the product’s attributes.

The glycan structures of therapeutic mAbs can play a pivotal role in determining drug effectiveness and safety [15]. They contribute to the stabilization of the CH2 domain of IgG, and the removal of glycosylation renders mAbs less thermally stable and more susceptible to unfolding. Furthermore, deglycosylated mAbs tend to aggregate [34]. According to the results of the glycan structure analysis, the percentages of fucosylated and mannosylated structures were similar to those of the reference. Research has revealed that afucosylated forms of human IgG1, constituting approximately 10% of normal human serum IgGs, are recognized as nonimmunogenic variants. Notably, afucosylated human IgG1 substantially augments antibody-dependent cellular cytotoxicity (ADCC). This enhancement is primarily attributed to an increased binding capacity to FcγRIIIA, and notably, no discernible modifications are observed in terms of complement-dependent cytotoxicity or antigen binding capacity [22]. Given that high-mannose-type glycans in the Fc region have been documented to exert a substantial influence on the circulating half-life of therapeutic antibodies, monitoring and modulating this factor could prove invaluable in managing the pharmacokinetics of mAbs [14].

The hydrophobicity of antibodies is an important factor, as it is correlated with their stability and predisposition to aggregation. In the resulting chromatograms of HIC analysis, prepeaks indicate the presence of truncated forms resulting from proteolytic cleavage. The main peak comprises a notably pure and biologically active mAb, whereas the postpeaks are heterogeneous and contain misfolded products alongside other impurities linked to the manufacturing process. These misfolded mAbs are generated early in the cell culture process and increase as the culture temperature rises (Fung, 2007). According to Fekete et al., [11], the postpeaks can be the mAbs that are associated with the CHO host cell proteins during the production process or a dimer peak. However, the SEC results demonstrated that there were no aggregates in any of the clones (Fig. 4). Therefore, the small peak in the HIC chromatogram may indicate a post translational modification. Importantly, while HIC is well suited for tracking oxidation, its effectiveness in detecting deamidation and isomerization is notably lower than that of ion exchange chromatography (IEX) [24].

High-affinity antibody‒antigen interactions are crucial in the biosimilar production process. Ramucirumab specifically binds to VEGFR-2 and prevents the binding of VEGF-A, VEGF-C, and VEGF-D [9]. The binding affinity coefficient values obtained from our SPR analysis are consistent with the value (Kd = 50 pM) reported in the literature [29]. Therefore, three of the four mAbs produced in our study had comparable binding affinities with the reference mAb. One of the mAbs exhibited a higher binding affinity to the antigen, whereas two of them showed similar binding affinities as the reference.

This study represents an early-stage proof-of-concept for a Ramucirumab biosimilar candidate. The work was performed at shake-flask scale and does not include bioreactor-based evaluation, which limits conclusions regarding process scalability. Downstream processing was limited to Protein A capture, and impurity characterization was therefore outside the scope of this study. In addition, functional assessment was based solely on SPR binding kinetics, without cell-based potency assays. Despite these limitations, this study provides a solid foundation for the development of a Ramucirumab biosimilar candidate. Future studies will focus on scale-up, full downstream processing, and expanded functional and impurity analyses to further advance and validate its biosimilarity.

## Conclusion

In this study, we outlined biosimilar development process from gene to product in CHO cells, highlighting the influence of process parameters on cell behavior and productivity. We achieved titers up to 2.79 g/L and demonstrated a high degree of structural similarity among selected producer clones. Our findings emphasize not only the feasibility of biosimilar production under well-monitored upstream conditions but also the importance of integrating process data with product analytics to enhance decision-making and ensure regulatory compliance. The produced antibody represents a promising early-stage biosimilar candidate with preliminary analytical similarity to the reference product. Future work should focus on bioreactor scale-up, implementation of downstream polishing strategies, comprehensive impurity clearance assessment, inclusion of mechanism-of-action–relevant cell-based potency assays, and forced degradation studies to support full biosimilarity evaluation.

## Supplementary Information

Below is the link to the electronic supplementary material.

Supplementary Material 1

## Data Availability

The data generated within the scope of the study are included in this published article and its supplementary information file.
