# Supplementary material for: Development and preliminary physicochemical characterization of a Ramucirumab biosimilar candidate in CHO cells using shake-flask fed-batch culture
Source: Bioprocess Biosyst Eng. 2026 May 27;49(8):2033–43. doi: 10.1007/s00449-026-03358-y (PMC13424466; doi:10.1007/s00449-026-03358-y)
Supplement: Supplementary file 1 — Supplementary Material 1 [file 449_2026_3358_MOESM1_ESM.docx]

**Development and Preliminary Physicochemical Characterization of a Ramucirumab Biosimilar Candidate in CHO Cells Using Shake-Flask Fed-Batch Culture**

Elcin CAGATAY^1,2^, Yonca GUNGOR^1,2^, Sadettin Seyit OZTURK^3^, Hulya AYAR KAYALI^1,2,4*^

^1^ Izmir International Biomedicine and Genome Institute, Dokuz Eylul University, Izmir, Turkey

^2^ Izmir Biomedicine and Genome Center, Izmir, Turkey

^3^ OzBio LLC, Dedham, MA, USA

^4^ Department of Chemistry, Faculty of Science, Dokuz Eylul University, Izmir, Turkey

***** Corresponding author email: [hulya.kayali@deu.edu.tr](mailto:hulya.kayali@deu.edu.tr) , +90 533 090 32 03

**SUPPLEMENTARY MATERIALS**

1. **Supplementary Methods**

**1.1. Gene Sequences of Ramucirumab**

***Heavy Chain***

EVQLLESGGGVVQPGRSLRLSCAASGFTFSSYGMHWVRQAPGKGLEWVAVISYDGSNKYYADSVKGRFTISRDNSKNTLYLQMNSLRAEDTAVYYCAKDMGWGSGWRPYYYYGMDVWGQGTTVTVSSASTKGPSVFPLAPSSKSTSGGTAALGCLVKDYFPEPVTVSWNSGALTSGVHTFPAVLQSSGLYSLSSVVTVPSSSLGTQTYICNVNHKPSNTKVDKKVEPKSCDKTHTCPPCPAPELLGGPSVFLFPPKPKDTLMISRTPEVTCVVVDVSHEDPEVKFNWYVDGVEVHNAKTKPREEQYNSTYRVVSVLTVLHQDWLNGKEYKCKVSNKALPAPIEKTISKAKGQPREPQVYTLPPSRDELTKNQVSLTCLVKGFYPSDIAVEWESNGQPENNYKTTPPVLDSDGSFFLYSKLTVDKSRWQQGNVFSCSVMHEALHNHYTQKSLSLSPGK

***Light Chain***

ELQMTQSPSSLSASVGDRVTITCRTSQSISSYLNWYQQKPGQPPKLLIYWASTRESGVPDRFSGSGSGTDFTLTISSLQPEDSATYYCQQSYDIPYTFGQGTKLEIKRTVAAPSVFIFPPSDEQLKSGTASVVCLLNNFYPREAKVQWKVDNALQSGNSQESVTEQDSKDSTYSLSSTLTLSKADYEKHKVYACEVTHQGLSSPVTKSFNRGEC

**1.2. ELISA**

Wells were coated with 1 µg/mL AffiniPure Fcγ fragment specific goat anti human IgG (Jackson ImmunoResearch, Cat. No. 109005008) diluted in sodium bicarbonate carbonate buffer (pH 9.6) and incubated overnight at 4°C. Wells were blocked with 1% bovine serum albumin in PBS containing Tween. Samples and standards were applied at appropriate dilutions and incubated for 1 hour at room temperature. After washing, HRP conjugated goat anti human IgG secondary antibody (Thermo Fisher Scientific, Cat. No. H10307) was added. TMB substrate was introduced, and the reaction was stopped with 0.18 M sulfuric acid. Absorbance was measured at 450 nm using a Multiskan FC Microplate Photometer.

**1.3. Protein Quantification**

***Bicinchoninic acid  (BCA) Assay***

The protein amounts in the purified mAbs were assessed also by Pierce BCA Protein Assay Kit (Thermo Fisher Scientific, Waltham, MA) according to the manufacturer's instructions. Bovine gamma globulin (BGG) was used as the calibration standard, and absorbance was measured at 562nm for colorimetric quantification.

***UV280 Measurement***

Antibody concentration after Protein A purification was determined by UV absorbance at 280 nm using a standard curve generated with purified monoclonal antibody.

**1.4. Sodium Dodecyl Sulfate‒Polyacrylamide Gel Electrophoresis (SDS‒PAGE) Analysis**

Cyramza was used as reference (Eli Lilly and Company, Cat: D396901). The samples were run on 8–12% SDS‒PAGE gradient gels under both reducing and nonreducing conditions. Reducing conditions included 40 mM dithiothreitol (DTT). Loading dye included 4 mM urea. All the samples were incubated at 70°C for 10 minutes prior to loading into the gel.

**1.5. Intact Mass Analysis**

A Xevo G2-XS QTof mass spectrometer (Waters, UK) with an electrospray ionization (ESI) source running in positive ion mode was used to perform intact mass analysis. Direct infusion was used to introduce the samples. Data were collected at a scan time of 1.0 s over a m/z range of 500–4000. The device was run with a cone voltage of 80 V, a capillary voltage of 3.0 kV, and a source temperature of 125 °C. A desolvation gas flow rate of 800 L/h and a desolvation temperature of 350 °C were used.

For intact mass determination of reference and produced mAbs, a Waters ACQUITY H-Class Bio UPLC system equipped with an ACQUITY UPLC BEH300 C4 column (2.1 mm × 50 mm, 1.7 µm) was used. Mobile phases containing 0.1% formic acid in acetonitrile and 0.1% formic acid in ultrapure water were used in the analysis. The elution was monitored at 280 nm via UV detection.

**1.6. Size-Exclusion Chromatography (SEC)**

SEC analysis was carried out using an Acquity H-Class Bio-UPLC instrument (Waters Corporation, Milford, MA). Different buffer compositions, pH values, column temperatures, and flow rates were optimized on a Protein BEH SEC column (200 Å, 1.7 µm, 4.6 mm × 300 mm) (Waters Corporation, Milford, MA). The analysis was performed using 100 mM sodium phosphate (pH 6.8) buffer under isocratic conditions at a flow rate of 0.3 mL/min and measured at a wavelength of 280 nm.

**1.7. Cation Exchange Chromatography (CEX)**

The charge variants of the reference and produced mAbs were analyzed via a Protein-Pak Hi Res SP, 7 µm, 4.6 × 100 mm column (Waters Corporation, Milford, MA) equilibrated at 30°C and operated at a flow rate of 0.3 mL/min. The mobile phases consisted of 100 mM MES monohydrate (pH 6.0) (mobile phase A) and 100 mM MES monohydrate containing 1 M NaCl (pH 6.0) (mobile phase B). The column was equilibrated with 30% mobile phase A and 70% ultrapure water. A gradient was applied by increasing mobile phase B from 0% to 30% over 10 minutes, while maintaining ultrapure water at 70%. The system was then returned to initial conditions at 11 minutes and re-equilibrated until 15 minutes. The elution was monitored by UV absorbance at 280 nm.

**1.8. Peptide Mapping Analysis**

For peptide mapping, 100 µg of reference and produced mAbs were filtered through a 10 kDa membrane in 0.1% Rapigest SF (Waters Corporation, Cat: 186008089). Retained proteins were recovered with 50 mM ammonium bicarbonate and denatured at 80°C for 15 min. Disulfide bonds were reduced with DTT (60°C, 15 min) and alkylated with iodoacetamide (RT, dark, 30 min). Digestion was performed overnight at 37°C using 1 µg/µl MS-grade trypsin. The reaction was quenched with TFA and incubated at 37°C for 20 min. After centrifugation, the supernatant was injected into UPLC-UV-Q-ToF following dilution in water/acetonitrile/formic acid.

**1.9. Glycosylation Assessment**

Glycan analysis was performed using the GlycoWorks RapiFluor-MS kit (Waters, Cat: 176003713) per the manufacturer’s protocol. mAbs (2 mg/mL) were denatured with RapiGest SF and deglycosylated with Rapid PNGase F (50°C, 5 min). Released glycans were labeled with RapiFluor-MS at room temperature. Samples were loaded into 96-well plates pre-equilibrated with 18:85 (v/v) water/acetonitrile and washed with 1:9:90 (v/v/v) formic acid/water/acetonitrile. Glycans were eluted with 200 mM ammonium acetate in 5% acetonitrile and analyzed on an ACQUITY Premier Glycan BEH Amide 130 Å, 1.7 μm, 2.1 × 150 mm column using 50 mM ammonium formate (pH 4.4) and acetonitrile as mobile phases (60°C, 0.4 mL/min, EX 330/EM 420 nm).

**1.10. Hydrophobic Interaction Chromatography (HIC)**

HIC analysis was performed on an Agilent 1260 Infinity II instrument (Agilent Technologies, Santa Clara, CA). Different buffers, pH values, column temperatures and flow rates were optimized for the MAbPac HIC-10 5 µm 1000 Å 4.6 × 100 mm column (Thermo Fisher Scientific, Waltham, MA). The optimized mobile phase A was 20 mM sodium phosphate pH 7.0 with 2 M ammonium sulfate, and mobile phase B was 20 mM sodium phosphate pH 7.0 buffer. A linear gradient was applied from 20% to 100% mobile phase B over 28 minutes (from 1 to 29 min), followed by a hold at 100% B from 29 to 34 min. The system was then re-equilibrated to initial conditions (20% B) from 35 to 40 min. The column temperature was set to 30^o^C. The analysis was carried out at 220 nm with a 0.5 mL/min flow.

**1.11. Surface Plasmon Resonance (SPR)**

The VEGFR-2 ligand (R&D Biosystems, Cat.No. 357-KD- 050/CF) was immobilized on a CM5 chip surface via amine coupling to approximately 500 response units. The surface of the sensor chip was activated with an amine coupling kit (Cytiva, Cat: BR100050). The ligand was diluted to 250 nM with 10 mM sodium acetate (pH 5.5). mAbs were subsequently injected over the ligand surfaces, and the binding kinetics were calculated via Biacore T200 evaluation software.

1. **Supplementary Results**

**
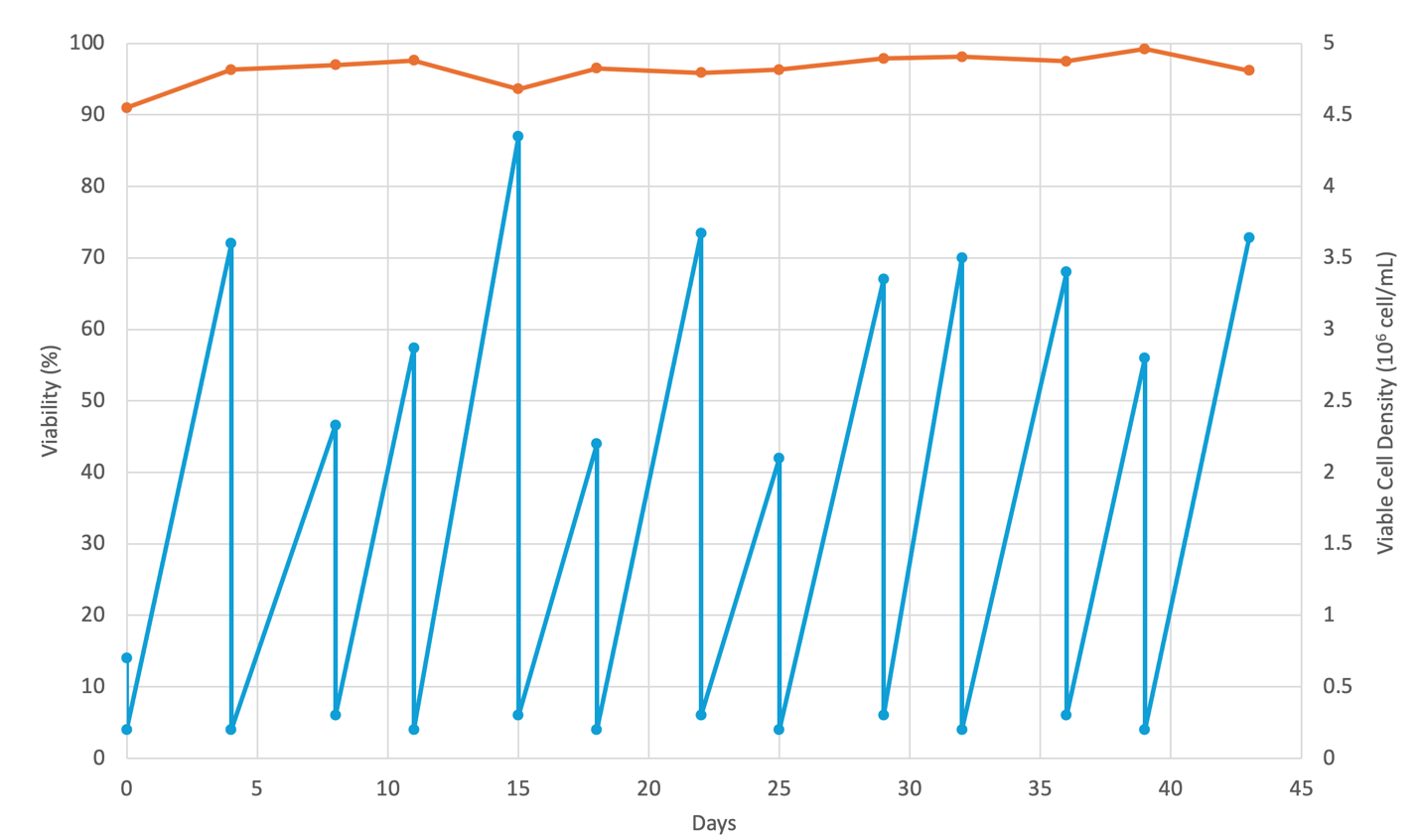
**

**Figure S1**. Growth profile of host CHO K1 cells.

**
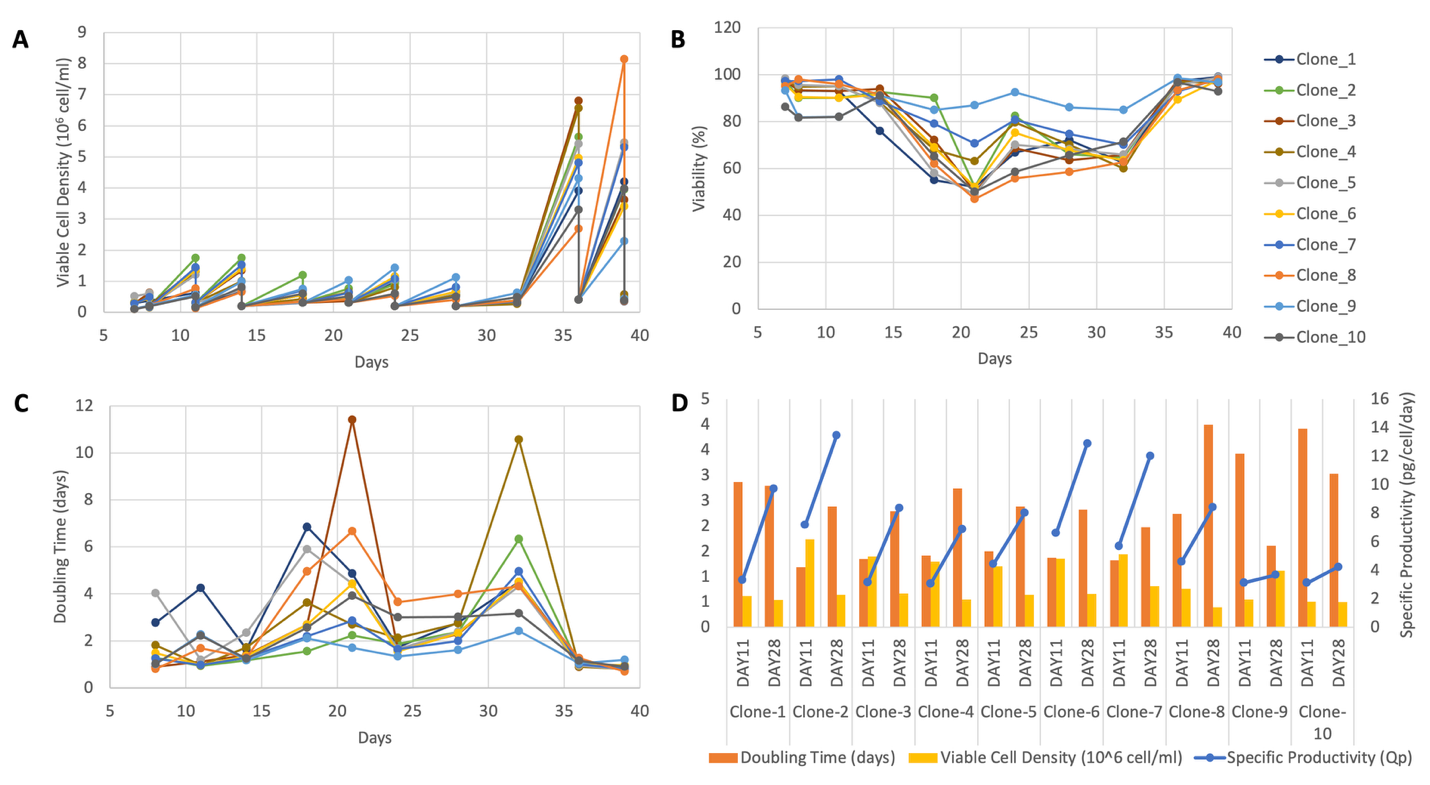
**

**Figure S2.** Growth and the productivity profiles of the single cell clones. (A) Viable cell densities, (B) viabilities, (C) doubling times, (D) changes of specific productivity of the single cell clones with doubling times and VCDs in between day 11 and day 28.


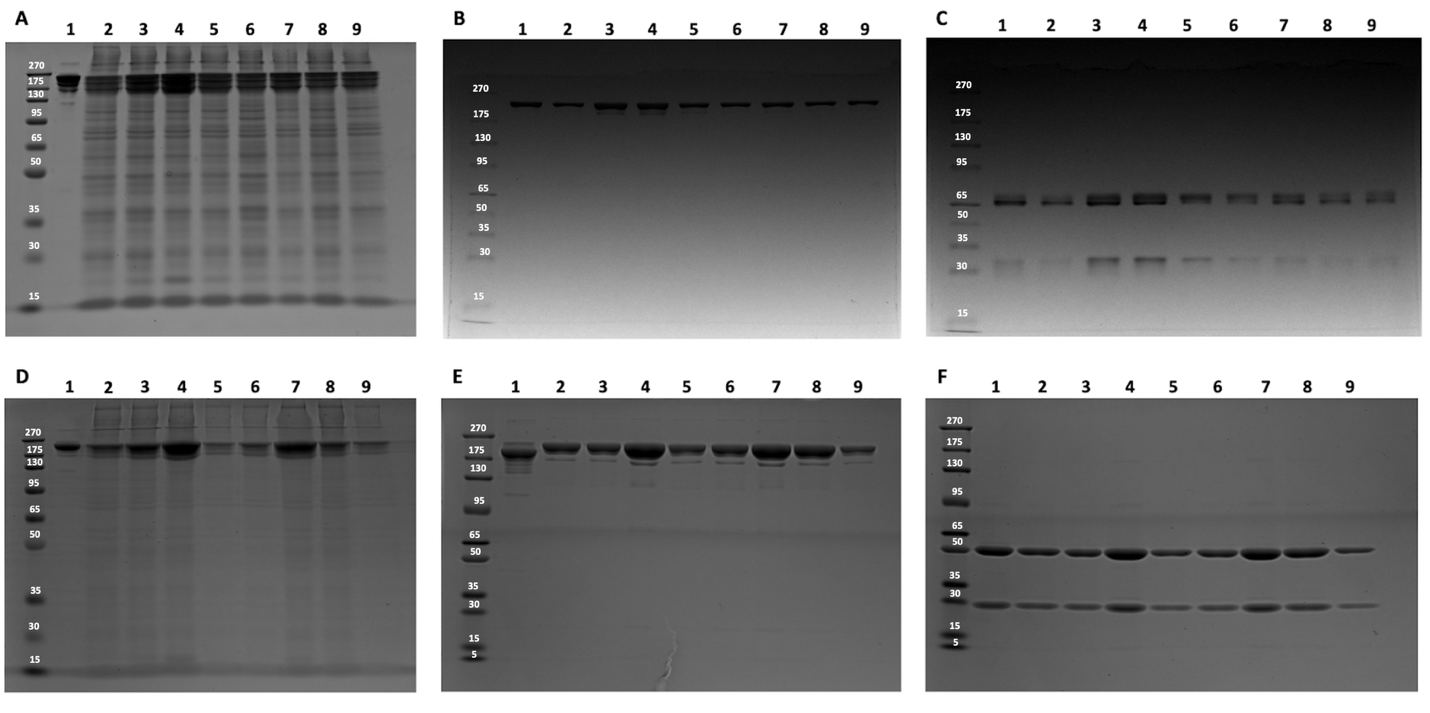


**Figure S3.** The supernatants of the best producing eight clones (lanes 2-9 respectively in each SDS PAGE image) were collected from the fedbatch cultures and analyzed for the comparison with the reference product (lane 1). (A) Intact SDS PAGE for the samples obtained from the cultured with GM_A media, before purification. (B) Intact SDS PAGE for the samples obtained from the cultured with GM_A media, after purification. (C) Reduced SDS PAGE for the samples obtained from the cultured with GM_A media, after purification. (D) Intact SDS PAGE for the samples obtained from the cultured with GM_B media, before purification. (E) Intact SDS PAGE for the samples obtained from the cultured with GM_B media,after purification. (F) Reduced SDS PAGE for the samples obtained from the cultured with GM_B media,after purification.


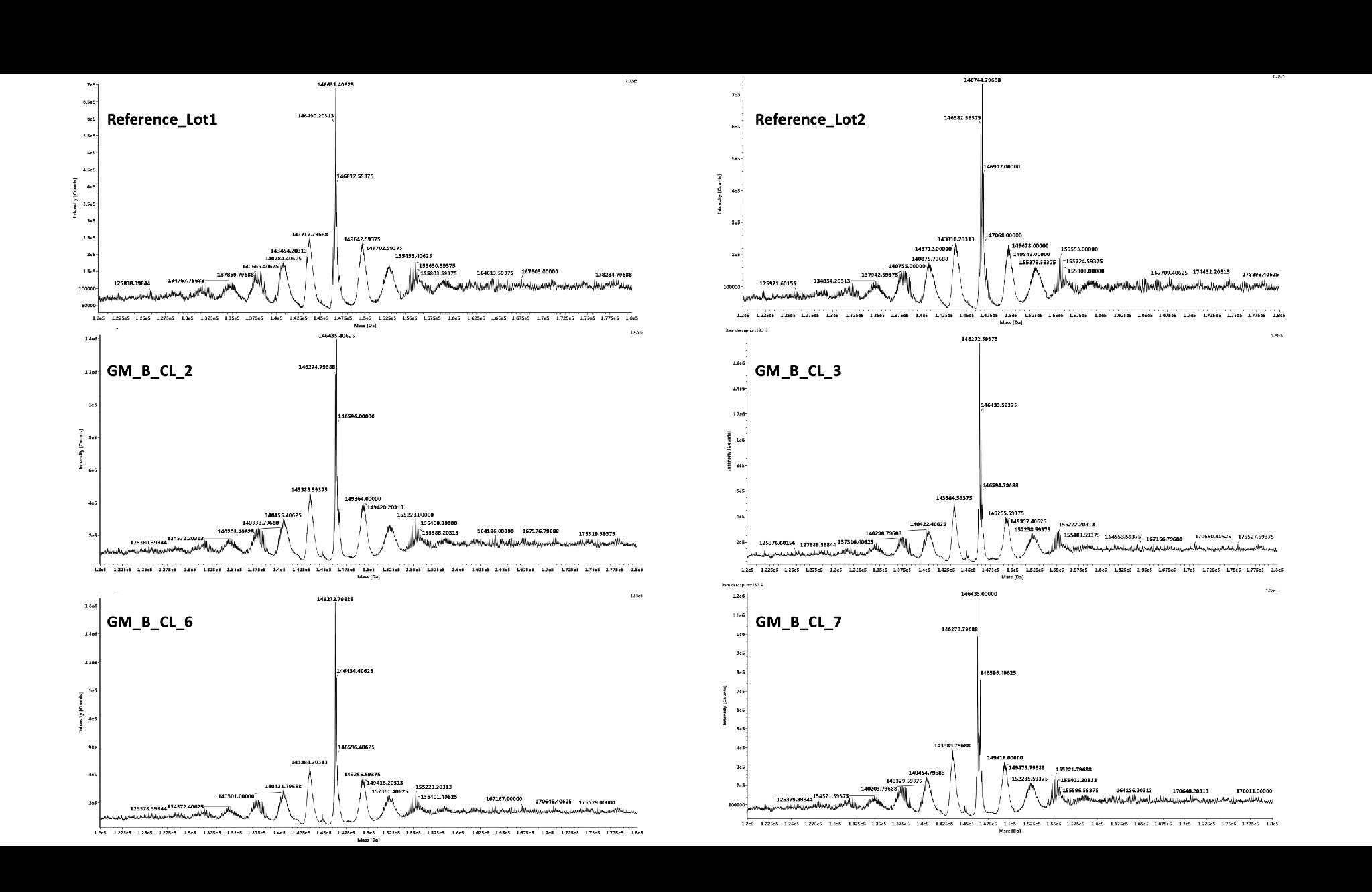


**Figure S4.** Deconvolved spectra of reference lots and GM_B clones.

**Table S1.** mAb titers (g/L) after Fed-Batch culture determined by ELISA.

| **GM_A_CL_1** | **GM_A_CL_2** | **GM_A_CL_3** | **GM_A_CL_4** | **GM_A_CL_5** | **GM_A_CL_6** | **GM_A_CL_7** | **GM_A_CL_8** |
| --- | --- | --- | --- | --- | --- | --- | --- |
| 1.07 ± 0.01 | 1.27 ± 0.09 | 1.42 ± 0.06 | 1.18 ± 0.03 | 0.94 ± 0.01 | 1.16 ± 0.02 | 1.07 ± 0.01 | 1.05 ± 0.02 |
| **GM_B_CL_1** | **GM_B_CL_2** | **GM_B_CL_3** | **GM_B_CL_4** | **GM_B_CL_5** | **GM_B_CL_6** | **GM_B_CL_7** | **GM_B_CL_8** |
| 1.34 ± 0.04 | 1.50 ± 0.1 | 2.4 ± 0.13 | 0.92 ± 0.01 | 1.22 ± 0.01 | 1.9 ± 0.16 | 1.42 ± 0.05 | 1.07 ± 0.01 |

**Table S2.** mAb titers of GM_A and GM_B samples after purification determined by BCA and UV280.

| **Concentrations (g/L)** | | | | | |
| --- | --- | --- | --- | --- | --- |
| **Clone** | **BCA** | **UV280** | **Clone** | **BCA** | **UV280** |
| **GM_A_CL_1** | 0.54 ± 0.006 | 0.45 ± 0.005 | **GM_B_CL_1** | 0.97 ± 0.008 | 0.78 ± 0.006 |
| **GM_A_CL_2** | 1.10 ± 0.033 | 0.94 ± 0.008 | **GM_B_CL_2** | 1.50 ± 0.013 | 1.27 ± 0.008 |
| **GM_A_CL_3** | 1.43 ± 0.006 | 1.29 ± 0.007 | **GM_B_CL_3** | 2.79 ± 0.056 | 2.64 ± 0.017 |
| **GM_A_CL_4** | 0.91 ± 0.014 | 0.71 ± 0.003 | **GM_B_CL_4** | 0.53 ± 0.005 | 0.35 ± 0.002 |
| **GM_A_CL_5** | 0.59 ± 0.008 | 0.52 ± 0.004 | **GM_B_CL_5** | 0.79 ± 0.007 | 0.59 ± 0.005 |
| **GM_A_CL_6** | 0.74 ± 0.011 | 0.68 ± 0.004 | **GM_B_CL_6** | 2.46 ± 0.061 | 2.17 ± 0.011 |
| **GM_A_CL_7** | 0.69 ± 0.022 | 0.56 ± 0.004 | **GM_B_CL_7** | 0.92 ± 0.020 | 0.78 ± 0.004 |
| **GM_A_CL_8** | 0.63 ± 0.10 | 0.51 ± 0.003 | **GM_B_CL_8** | 0.56 ± 0.011 | 0.4 ± 0.003 |

**Table S3.** The Kd values of reference and sample mAbs for binding to VEGFR-2 ligand.

|  | **Reference Range** | **GM_B_CL2** | **GM_B_CL3** | **GM_B_CL6** | **GM_B_CL7** |
| --- | --- | --- | --- | --- | --- |
| **KD (pM**) | 10.55 – 32.56 | 29.67 | 7.175 | 34.95 | 82.37 |
